# Medical ozone on hamstring injury in a professional athlete assessed by thermography: a clinical case report

**DOI:** 10.1259/bjrcr.20220078

**Published:** 2023-06-13

**Authors:** Francisco Javier Hidalgo-Tallón, Rosa Pinto-Bonilla, Jose Baeza-Noci, Silvia Menéndez-Cepero, Alessio Cabizosu

**Affiliations:** 1 Chair of Ozone Therapy and Chronic Pain, San Antonio Catholic University of Murcia (UCAM), Institute of Neurosciences, University of Granada, Granada, Spain; 2 Faculty of Medicine, University of Valencia, Valencia, Spain; 3 National Centre for Scientific Research, Havana, Cuba; 4 THERMHESC Group, Chair of Ribera Hospital de Molina - San Antonio Catholic University of Murcia (UCAM), Molina de Segura, Spain

## Abstract

Injuries associated with the hamstring muscles in the running athlete are increasingly investigated due to the economic and functional consequences associated with them. Although hardly used in the treatment of sports injuries, medical ozone is effective and very well tolerated in the treatment of musculoskeletal pain, it was decided to add a series of medical ozone infiltrations to the treatment. The evolution of the case was recorded by medical thermography, in addition to measuring pain intensity (visual analog scale) and functional capacity (toe touch test). Pain intensity (visual analog scale) decreased from seven at baseline to two at the end of treatment (after two ozone infiltrations, one weekly). Mobility of the damaged area (toe touch test) improved from a distance of 8 cm at baseline to 0 cm at the end of treatment. Regarding medical thermography, after the first and second infiltration of ozone, the temperature rose to a significant increase in perfusion from baseline from 31.2 to 31.8 °C and from 31.2 to 32 °C, respectively. These results suggest the possible interest of medical ozone as an adjuvant treatment for the recovery of sports tendinopathies and encourage us to carry out further studies.

## Introduction

Muscle tendon injuries of the proximal lower body, and more specifically the hamstrings, are very common in athletes.^1–3^ If not treated correctly, they can become chronic and compromise the functions and capacity of the affected structures, impairing performance. One of the sports fields most exposed to this type of injury, with major economic and functional repercussions, is running, where research has been intensified in recent years in the diagnosis, treatment, and follow-up of muscle-tendon problems.^4,5^

We consider medical ozone as a therapeutic element in those pathologies with hypoxia, inflammation and redox imbalance.^6^ Medical ozone is a mixture of medical oxygen and ozone at a specific concentration using an approved ozone device. More and more health professionals are using medical ozone in the treatment of musculoskeletal pain,^7,8^ especially in lumbosciatica^9–11^ and arthritic knee pain^12,13^ where the levels of evidence are high. Ozone therapy has been tested in a wide variety of inflammatory pathologies of the musculoskeletal system, both pre-clinical and clinical^14^ including various tendinopathies.^15,16^

Clinical thermography is a diagnostic technique that allows us to record images according to the emission of infrared radiation from a body surface, thus being able to distinguish changes linked to different physiological states, especially related to vascular flow.^17,18^ Changes in thermographic measurements have been correlated with clinical and vascular improvements or worsening. Recent studies highlight the reliability and sensitivity of this technique concerning the muscular and tendon system.^19–21^ However, we have not found any work that uses thermography to evaluate functional and pain changes after medical ozone treatment.

To study the therapeutic effects of medical ozone on a hamstring tendon injury in a professional sportswoman, it was decided to make a prospective record of the functional evolution and thermographic images before, during and after a series of applications of infiltrated medical ozone. The clinical case and its evolution are described below.

## Case presentation

A professional athlete in the 3.000 m steeplechase race, female, 28 years old, 160 cm tall and weighing 49 kg. In her usual training, the patient routinely carries out strength and endurance training sessions at least six times a week, with an average duration of 2–3 h per session.

The patient came to our clinic with pain at rest in the back of the right thigh, 3 weeks in duration and intensity 7 out of 10 according to the visual analog scale (VAS).^22^ To the baseline record on the day of the initial examination, another record would be added each week, before each infiltration session.

She had previously been prescribed a conventional treatment consisting of relative rest, a warming cream three times a day, based on menthol and essential oils (red tiger balm), and analgesic-anti-inflammatory medication (ibuprofen 600 mg every 8 h, paracetamol 1 g every 8 h and diclofenac sodium 75 mg intramuscularly daily), with very limited success.

Exploring the proximal myotendinous junction of right semi-membranosus area, the pain increased to intensity 9. No pain was palpable on pressure over the ischium (discarding clinical periostitis), and no pain was felt towards the distal portion of the muscle belly, suggesting a limited lesion. Every visit, the patient would be examined by palpation over the painful area and the associated musculature, looking for tight bands and trigger points.

A tight band was noted along with the hamstring musculature, presumably linked to a limitation of range of motion according to the toe touch test.^23–25^

This test was used to assess functional limitation, presumably due to the injury and/or related myofascial syndrome, as it measures the flexibility of the hamstring muscle complex. The patient, starting from a bipedal position, performs a forward trunk tilt with hands and fingers extended. The examiner measures the distance between the ends of the fingers and the ground.

The Samsung HS-70A Ultrasonography revealed a proximal partial tear in the right semitendinosus tendon, which was confirmed by MRI.

It was decided to carry out a treatment consisting of a series of infiltrations with medical ozone that would be performed weekly around the injured area, depending on the evolution of the condition. The treatment course was explained to the patient, as well as the basics of infiltrated medical ozone and thermography. All drug treatment was suspended.

After taking the necessary measurements and disinfecting the skin surface with topical chlorhexidine spray (10 mg ml^−1^), the lesion was treated by ultrasound-guided infiltration with 10 ml of medical ozone at a concentration of 20 µg ml^−1^. The punction was carried out in the proximal myotendinous junction of the hamstring musculature, around the right semi-tendinosus tendon. Before each ozone infiltration, the site was superficially anesthetized with 1 ml of 3% subcutaneous mepivacaine.

The infiltrated ozone was obtained from a medical ozone generator with European IIB approval, model Ozonete, developed by the Spanish Society of Electromedicine and Quality S.A. (SEDECAL-Sociedad Española de Electromedicina y Calidad S.A.), Madrid, Spain.

The number of sessions to be undertaken would depend on their efficacy and tolerability and would be carried out weekly, establishing a maximum of 6 weeks of treatment, following our clinical experience.

The patient signed the informed consent for the first session both for the treatment and for sharing and publishing her medical data for scientific purposes, following the requirements of the Thermographic Imaging in Sports and Exercise Medicine checklist, which is a proposal to standardize data collection and analysis and ensure good clinical practice in the technique.^26^

Thermographic recordings would be made (both on the injured and contralateral sides) before each infiltration (baseline), immediately afterward, and after 45 min, which was considered enough time to record the changes due to the effects of the infiltrations. Pain intensity measured by VAS would be recorded before the first infiltration (baseline) and weekly before each new session, thus avoiding contamination by punctures. The toe touch test would be measured before starting the treatment (baseline) and 10 min after each ozone infiltration, to assess the change in hamstring distensibility once the transient pain of the gas infiltration had ended.

The reliability of the technique is based on strict compliance with the Thermographic Imaging in Sports and Exercise Medicine protocol, a standardized and agreed protocol to avoid bias and to enhance the value of clinical thermography. According to this protocol, the patient could not sunbathe or use sunbeds from 1 week before the recordings. Nor could she do sport or any physical therapy for 24 h before the recording. In addition, from 2 h before the measurements, the patient would have to refrain from showering, applying cosmetics, eating, drinking caffeinated beverages or alcohol, smoking, or consuming medicines and narcotic substances.

Based on previous studies^27–29^ before the thermographic measurement, the patient would undergo a body temperature analysis with a digital thermometer to rule out basal temperature alterations, and the study could only be carried out if the basal temperature was less than 37°C. To acclimate to the environment, the patient would remain for 15–20 min in a room suitable for the study, 8 m^2^ in size, at an ambient temperature between 20 and 22°C, with a humidity of 30%, and an atmospheric pressure of 1 ATM. The room would be free of electronic devices to not interfere with the thermograph. The light would be warm and indirect on the patient.

Before proceeding with the thermographic recording of the posterior thigh, the patient was placed without trousers and in a standing position on a 1 cm thick base, located 1 m away from the thermograph placed on a tripod.

The thermograph used to carry out this work was the Flir E60 model, with thermal sensitivity <0.05°C and recordings from −20°C to +650°C. Due to this thermal sensitivity, changes exceeding 0.5°C are considered significant.

To correctly set up the machine, the same expert researcher would turn on the machine half an hour before the first recording, placing it at an angle of 10°/15° to the patient. The emissivity would always be 0.98, as suggested by the manufacturer and according to previous studies.^30–32^

The regions of interest (ROIs) were defined using anatomical references by placing an aluminum strip 5 cm below the ischial tuberosity, the point of pain described by the patient. The aim was to record changes in infrared emission at a distance and relate them to the anatomical area of interest. The ROIs were analyzed with Flirtools software^33^ .

## Results

After two consecutive sessions, the improvements recorded were remarkable, and the tolerability of the treatment was excellent, with the patient reporting to feel “really better”, without any secondary inflammatory reaction, which allowed her to resume her training.

According to the VAS records, the pain went from intensity seven before starting treatment (baseline) to intensity five, 1 week after the first session and intensity two, 1 week after the second session (Table 1).

**Table 1.** Evolution of patient

VAS SCALE						
Day 0 (Basal)	Day 7			Day 14		
7	5			2		
TOE TOUCH TEST						
Day 0 (pre-intervention)	Day 0 (10 min after ozone intervention)			Day 7 (10 min after ozone intervention)		
8 cm	3 cm			0		
THERMOGRAPHY TEST						
	Day 0			Day 7		
	Basal T	Anesthetic + ozone	45 min post- infiltration	Basal T	Anesthetic + ozone	45 min post- infiltration
Right	31.2°C	29.0°C	32.9°C	31.8°C	29.6°C	32.7°C
Left	32.0°C	31.6°C	32.1°C	32.1°C	31.8°C	32.0°C

The improvement was also significant according to the changes recorded in the toe touch test (Table 1), as the measurements went from a baseline distance of 8 cm to a distance of 3 cm after the first treatment session (Day 0) and to 0 cm after the second infiltration (Day 7).

According to the temperature of the treated area, there were significant changes in the defined ROIs (Table 1).

**Figure 1.**
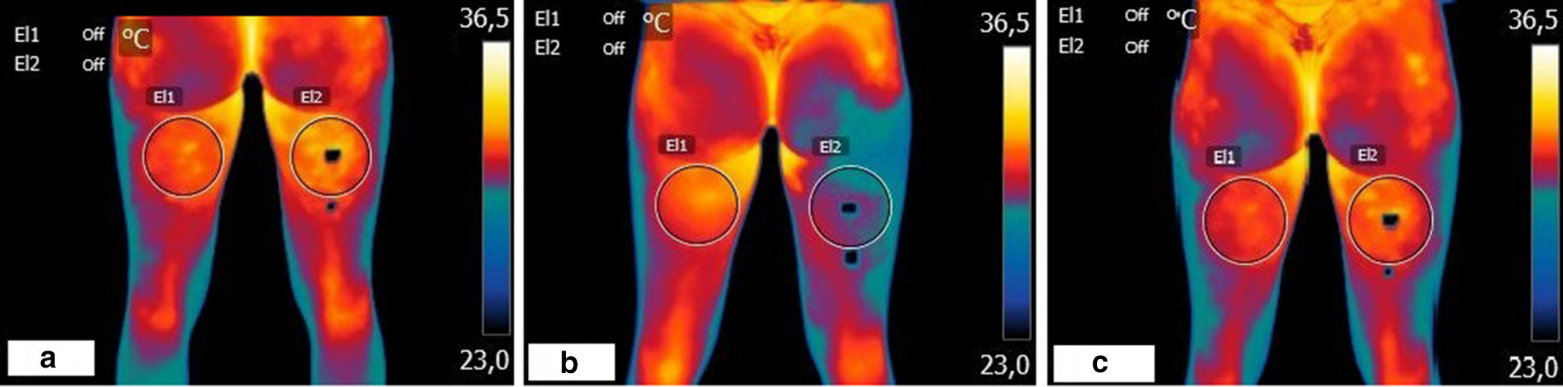
Thermographic images recorded in the first session (Day 0), (**a**) Basal,** (b**) immediately after infiltration,** (c**) 45 min after infiltration.

As expected, due to the inflammatory process of the lesion, in the baseline recordings there were significant changes in the temperature of both legs (32.0°C *vs* 31.2°C). Immediately after the first infiltration, there was a loss of temperature in the area, dropping from 31.2 to 29°C, probably due to the vasoconstrictive effect of the 3% mepivacaine. After 45 min, the temperature rose again in the area of the lesion, presumably due to the effects of ozone, reaching a significant increase in perfusion with respect to baseline (from 31.2 to 31.8 °C) (Figure 1).

**Figure 2.**
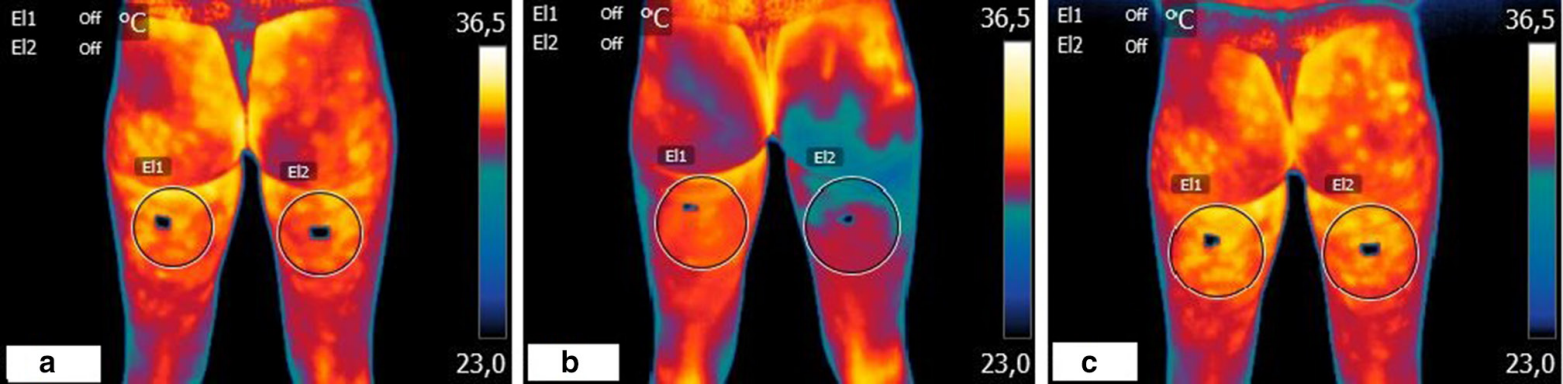
Thermographic images recorded in the second session (Day 7). (**a**) Basal,** (b**) immediately after infiltration,** (c**) 45 min after infiltration

Likewise, in the second session (Figure 2), there was almost immediate vasoconstriction after infiltration, lowering the temperature from 31.8 to 29.6°C, with delayed recovery of the temperature recorded 45 min later, marking significant differences with respect to baseline (from 31.2 to 32°C).

Remarkably, the largest differences were recorded between the values before the ozone sessions and those recorded 45 min after the second treatment session, with a rise in temperature from 31.2 to 32.7 °C.

Muscle-tendon injuries are frequent in sports practice and lead to interruptions in training and optimization schedules, especially affecting elite athletes. In the case presented here, a myotendinous injury to the right hamstring muscles of a professional runner who had not improved with conventional treatment was treated with medical ozone.

It was decided to use infiltrated medical ozone as an adjuvant for the treatment of the injury, prospectively recording the evolution of pain using VAS, biomechanical changes using the toe touch test, and hemodynamic and inflammatory changes using thermography.

The result was positive in a lesion that had not progressed with conventional treatments for 3 weeks. Both the pain intensity and the mobility of the injured area improved after the first application of medical ozone, as well as favorable changes were recorded in the thermographic values. The ozone sessions were perfectly tolerated.

Pain intensity changed from VAS seven at baseline to VAS five, 1 week after the first intervention, and to VAS two 15 days later, after two ozone therapy sessions. We suggest that the analgesic and anti-inflammatory effects of ozone are responsible for this improvement.

Functional capacity also improved, going (according to the toe touch test) from 8 cm at the initial measurement to 3 cm after the first infiltration and to 0 cm after the second. We believe that by improving pain, vascularization, and the local inflammatory reaction, functional capacity also improved.

Thermographic imaging also showed significant changes from baseline to the first and second treatment sessions (from 31.3 to 32.9 °C and 32.7°C, respectively). In both sessions, there was a decrease in vascularity immediately after infiltration, probably due to the immediate vasoconstrictive effect of the 3% mepivacaine^34–37^ but this state was transient, as 45 min after the local ozone injection, reactive vasodilatation occurs, which might be due to the medical ozone effect, and significantly improves heat uptake by the thermographic camera. Moreover, this effect is longlasting, as observed when comparing the data collected at baseline, after the first session and after the second session, 1 week later. Lipoperoxides produced by the ozonation of human plasma have been shown to induce nitric oxide synthase in endothelial cells, releasing nitric oxide and nitroso thiols, which improves blood supply, especially in ischemic areas.^38^

## Discussion

Infiltrated medical ozone has been used with great safety in the treatment of musculoskeletal pain since the 1980s.^39^ Its analgesic and anti-inflammatory efficacy is based on its oxidative capacity, which blocks biochemical soup (afferent silence), stimulates remyelination and tissue regeneration, improves local antioxidant capacity, inhibits pro-inflammatory cytokines, stimulates anti-inflammatory cytokines, and improves capillarity. The level of evidence as an analgesic, anti-inflammatory and restorative agent is high in the treatment of lumbosciatica and painful osteoarthritic knees, and the excellent tolerability of the technique is remarkable. In a recent study, Fernández Cuadros and his team recorded, without any adverse events, significant improvements in pain and stiffness in 115 patients with osteoarthritis of the knee using a protocol similar to the one described here, with four weekly infiltrations of medical ozone at a concentration of 20 µg ml^−1^.^40^

Initially, it was not possible to know how many sessions would be necessary. It was decided that these would be weekly to allow for the resolution of any inflammatory reactions, considered to be normal. However, in only two infiltrations the improvement was clear, and the patient was able to return to her sporting activities.

We did not find any essay in the literature about the use of ozone therapy in sports, however the use of infiltrated medical ozone has been described in a retrospective study published by Re and his team.^41^ This is a series of 183 athletes treated for various injuries (painful shoulder, low back pain, knee sprains, muscle tears and Achilles’s tendinitis). Most (85%) recovered quickly, especially those treated earlier, with improvements lasting 12–24 months. Treatments were well tolerated, reducing the requirement for anti-inflammatory drugs (steroidal and non-steroidal).

The same team reported success in a case resembling the one presented here, involving a right semi-membranosus injury in a professional athlete who received sessions of intramuscular medical ozone, twice-weekly, at concentrations between 8 and 12 µg ml^−1^, for 4 weeks. The treatment was effective, and no adverse effects were reported.^42^

There is considerable experience in the treatment of sports injuries with another oxidative therapy, hyperbaric oxygen therapy. The systemic biological effects of this technique are like those described for systemic ozone therapy (improvement of oxygen supply, phagocyte function, fibroblast activity, tissue repair, inflammation, infection and ischemia-reperfusion damage),^43^ and has demonstrated efficacy in the treatment of muscle, tendon and ligament injuries.^44,45^

Although quality studies are scarce and inconclusive, it seems that the greater availability of oxygen would favor the functional recovery of muscles and tendons-ligaments, accelerating the recovery of injured athletes. These circumstances would justify our work, in which we use another oxygenating, anti-inflammatory and regenerating therapy such as ozone therapy, which has demonstrated its efficacy and safety in the treatment of pain. Our results show an increase in post-treatment temperature in the injured area and a clinical improvement according to muscle stretching tests and pain records, which allowed the patient to return to sporting activity.

In the field of sport, the correlation between oxygenation, performance and pain has been extensively studied, and in muscle and tendon injuries the role of the vascularization plays a major role in the recovery process,^46^ Thermography has proven reliable as a method of quantifying local vascular changes that have been correlated with functional and pain improvements.^47,48^

Cabizosu et al have shown that in neuromuscular diseases, there is an inverse correlation between muscle atrophy and vascularization measured by thermography.^27–29^

Our work shows that before the first ozone therapy intervention there is less temperature, less elasticity, and more pain around the lesion. It is also clear that as pain and elasticity improve, the temperature on the affected side recovers, becoming similar to that of the healthy side.

Ozone therapy as an adjuvant could be useful for early recovery from sports injuries. We believe that it would be worth conducting open prospective studies and/or well-designed clinical trials, with adequate samples, to demonstrate the usefulness of this technique in sports medicine.

We would also like to highlight the role of thermography as a useful tool to evaluate vascularity (before and after medical ozone treatments) and correlate it with other clinical and functional parameters.

Injected medical ozone is effective and well-tolerated in the treatment of musculoskeletal pain, and could also be useful in the treatment of sports injuries and performance recovery. It is suggested that controlled studies must be carried out to study the contributions that this technique could add to conventional treatments.

## Learning points

- Thermography is a useful tool in the assessment of musculoskeletal injuries and ozone treatment.
- Ozone therapy can increase the vascularization of the tissues, favoring the recovery of the inflammatory process.
- We observe a relationship between the VAS scale and thermography in acute muscle damage.
